# The Grown in Wales Study: Examining dietary patterns, custom birthweight centiles and the risk of delivering a small-for-gestational age (SGA) infant

**DOI:** 10.1371/journal.pone.0213412

**Published:** 2019-03-12

**Authors:** Samantha M. Garay, Katrina A. Savory, Lorna Sumption, Richard Penketh, Anna B. Janssen, Rosalind M. John

**Affiliations:** 1 Biomedicine Division, School of Biosciences, Cardiff University, Cardiff, United Kingdom; 2 Department of Obstetrics and Gynaecology, University Hospital Wales, Cardiff, Wales, United Kingdom; University of Leeds, UNITED KINGDOM

## Abstract

**Objectives:**

Maternal lifestyles, including diet, have been linked to infant birthweight. However, customised birthweight centiles (CBWC), which more accurately identify small babies that have increased fetal growth restriction and are at higher risk of newborn morbidity and later life health complications, are rarely considered when studying maternal diet. This study investigated maternal dietary patterns and their impact on infant CBWC within a cohort of women living in South Wales.

**Methods:**

This study utilised cross-sectional data from the longitudinal Grown in Wales (GiW) cohort. Women 18–45 years old were recruited the morning prior to an elective caesarean section (ELCS). Women completed a food frequency questionnaire (FFQ). Additional data on pregnancy and birth outcomes was extracted from medical notes. Data from 303 participants was analysed.

**Results:**

‘Western’ and ‘Health conscious dietary patterns were identified. The ‘Health Conscious’ dietary pattern was significantly associated with maternal BMI, age, education, income and exercise. Adjusted regression analyses indicated that greater adherence to a ‘Health Conscious’ dietary pattern was significantly associated with increased CBWC (AOR = 4.75 [95% CI: 1.17, 8.33] *p* = .010) and reduced risk of delivering a small-for-gestational age (SGA) infant (AOR = .51 [95% CI: .26, .99] *p* = .*046*).

**Conclusion:**

A healthier diet was significantly associated with higher birthweight using CBWC and a reduced risk of delivering an SGA infant suggesting that birthweight will be improved in areas of Wales by focused support encouraging healthier dietary habits.

## Introduction

Diet in pregnancy, whether balanced and healthy or unhealthy, can have important implications for birth weight and later life health. In Wales recent statistics indicate that 11.4% of live births were classified as high birthweight (HBW) [1], with a general shift towards heavier babies [2]. Despite this general increase in birthweight, 6.9% of live births were classified as low birthweight (LBW), with no significant improvements in LBW prevalence since 2011 [1]. Moreover, the prevalence of low birthweight in live births varies by as much as 3.1% across health boards in Wales, which cover areas of varying socioeconomic status, highlighting disparities in improvements in birthweight [3]. Specific studies of Welsh populations are important due to the devolution of responsibility for the National Health Service to the Welsh Assembly Government in 1999 resulting in differences between the nations on vital areas such as health systems [4], health priorities and health outcomes[5]. Consequently, it cannot be assumed that outcomes will be the same across nations.

Traditionally, infants are classified as HBW if birthweight is 4kg or above and LBW if below 2.5kg [1]. LBW can be a consequence of a premature birth or fetal growth restriction (FGR) where the baby has not reached its genetic growth potential, or both factors [6], with different causes and consequences. However, issues have been highlighted with the traditional birthweight classification system, such as they cannot distinguish between infants born physiologically or pathologically small and utilise arbitrary cut-offs ensuring there is little difference between an infant born 2.49kg and 2.5kg, despite being classified as LBW and normal birthweight respectively [7, 8]. Custom birthweight centiles (CBWC) have been designed to address these issues and are calculated by adjusting for factors that may affect fetal growth; maternal height, weight, parity, ethnicity, gestational age and fetal sex. It is possible to classify infants according to their CBWC as small-for-gestational age (SGA), average-for-gestational age (AGA) or large-for-gestational age (LGA). LGA infants are those above the 90^th^ centile, whilst SGA infants are typically below the 10^th^ centile, with SGA also considered a proxy measure for FGR. As SGA is based on standardised centiles, it is different from the unstandardised traditional population-based classification of LBW [9]. Consequently, CBWC are able to distinguish between infants born pathologically rather than physiologically small [9, 10] and provides more accurate classifications of SGA [11], thus preventing pregnancies from undergoing unnecessary investigation and intervention [12].

SGA infants are at increased risk of neonatal morbidity [13] and mortality [14–16], adverse neurocognitive outcomes and development in childhood [17, 18], and longer term ill health including heart disease and related disorders of hypertension, stroke and diabetes [19]. Delivering an SGA baby is also associated with future risk of maternal cardiovascular disease and death [9, 20]. Conversely, LGA has been associated with increased risk of obesity in childhood [21, 22].

Research has indicated that various factors may be related to infant birthweight outcomes, including maternal diet. Typically, studies have investigated this area by examining individual dietary components [23]. However, diet is varied and rarely consists of single dietary components, thus this method cannot account for or address intercorrelations between food groups [24]. Dietary patterns overcome this limitation and have been examined in pregnancy in various regions of Europe, including the UK [25–27], Spain [28], Finland [29] and Norway [30]. Studies have also investigated the relationship between dietary patterns and infant birthweight outcomes. Healthier diets are generally associated with increased birthweight, and thus lower risk of SGA or FGR [31, 32]. Conversely, evidence has largely indicated that unhealthy diets are related to lower birthweight and increased risk of SGA [32, 33]. However, only one study has applied customised birthweight measures that account for various maternal and child characteristics to study maternal diet [31], as typically studies adjust for only gender and gestational age, although [34] did customise for ethnicity, crucial in a multiethnic cohort. Moreover, no studies have examined dietary patterns, alone or in relation to infant birthweight, for women living in Wales. The current study examined dietary patterns and the impact on infant birthweight outcomes via CBWC within the Grown in Wales (GiW) cohort.

## Materials and methods

### Participant recruitment

Full ethical approval for the study was obtained via the Wales Research Ethics Committee (REC), reference 15/WA/0004. The GiW cohort has previously been described in [35]. Briefly, the GiW cohort is a longitudinal study in the South Wales region of the United Kingdom, with women recruited and providing their written consent the morning prior to a booked elective caesarean section (ELCS) by research midwives at the University Hospital of Wales (UHW) between 1^st^ September 2015 and 31^st^ November 2016. Women were invited to participate in the study if it was a singleton pregnancy without fetal anomalies and infectious diseases. Biological samples were collected for a future study (maternal blood, maternal saliva, placenta and cord blood).

### Materials

The current study specifically utilised data gathered the morning prior to a booked ELCS and from medical notes. An extensive questionnaire was completed by consented participants that included questions on sociodemographics and lifestyle (smoking, alcohol consumption, exercise and diet). Infant and placental weights were recorded by the research midwife, and where possible data on the pregnancy and outcome was also extracted from maternity notes. CBWC were calculated via the GROW bulk centile calculator (UK) [36], utilising data collected on maternal height, weight, ethnicity and parity as well as infant birthweight, gender and gestational age. A food frequency questionnaire (FFQ) was specifically designed for the GiW study with information on diet collected on 17 items. Whilst this has not currently been formally validated, it was evaluated by a nutritionist with extensive practical experience of the typical diet of pregnant women living in Cardiff, to ensure that a sufficient range of food items was included on the basis of which diet could be simply assessed. The FFQ was reviewed for suitability by 10 women who were either pregnant or had recently given birth and then piloted with 289 pregnant participants. The FFQ required participants to rate the frequency of consumption of the various food items since becoming aware of the pregnancy. The frequency was rated on a five point scale as ‘never/rarely’, ‘once in 2 weeks’, ‘2–3 times per week’, ‘once per day’ and ‘more than once per day’.

### Participants

Initially, 355 women were recruited to the GiW study, of which seven withdrew. The study focused on Caucasian participants to obtain a homogenous local cohort sample, who were 37 weeks gestation or above. Data from these 312 women was considered for inclusion in the current study. Participants missing five or more responses on the FFQ were excluded from the analysis. For those missing four or less responses, the missing responses were entered as ‘never/rarely’. This left 303 women for the current analysis.

### Statistical analysis

All analyses were undertaken utilising IBM SPSS Statistics Version 23. Dietary patterns were established through factor analysis, specifically principal component analysis (PCA) with orthogonal varimax rotation. Dietary pattern scores were generated for each participant to enable further analysis. Associations between variables and dietary patterns were identified via univariate and multiple linear regression, with those significant at the 0.05 level in the univariate analysis included in a multiple linear regression, after assessing for multicollinearity via tolerance and VIF scores. To investigate associations between dietary patterns and CBWC, linear regression was again utilised, with models both unadjusted and adjusted for potential confounders identified from existing literature. As CBWC already controlled for various maternal characteristics as well as gestational age, these potentially confounding variables were maternal age, smoking during pregnancy, alcohol during pregnancy, exercise, gestational diabetes mellitus (GDM), conception and Welsh Index of Multiple Deprivation (WIMD) score. To investigate the relationship further, separate unadjusted and adjusted binary logistic regressions were also utilised to investigate the associations between dietary patterns and SGA compared to AGA and LGA compared to AGA, defined as < 10^th^ centile and > 90^th^ centile accordingly. Associations were considered significant if *p* < 0.05 and no data transformations took place prior to analysis. A post-hoc power calculation was conducted utilising G*Power (version 3.1.9.2) [37] to determine the suitability of the sample size in the main analysis investigating dietary patterns and CBWC, which utilised linear regression. Effect size (*f*^2^) was set at .15 by utilising *r*^*2*^ = .130 from the analysis, and α error probability set at .05. This determined that n = 303 was sufficient to obtain statistical power > 95%.

## Results

### Demographics

Following the exclusion criteria, data of 303 participants from the 355 recruited to the GiW cohort was utilised in the current analysis. Table 1 displays the demographic data for these participants. Categorical data are expressed as % (n) and continuous data as median (IQR). A comparison of demographic data for participants included and excluded from the study, with the exception of those participants who withdrew, can be found in S1 Table.

**Table 1:** Demographic data of the 301 participants in the analysis.

Demographics	% (n) or median (IQR)
Maternal BMI at booking	26.17 (7.42)
Maternal age at booking	33.00 (6.00)
Parity, *% (n)*	
Multiparous	79.50 (241)
Nulliparous	20.50 (62)
Gestational weight gain (kg)	14.85 (7.88)
GDM	
Yes	95.00 (285)
No	5.00 (15)
Fetal sex, *% (n)*	
Female	54.10 (164)
Male	45.90 (139)
CBWC	58.60 (48.90)
Size for gestational age *% (n)*	
SGA	7.30 (22)
AGA	78.20 (237)
LGA	14.50 (44)
Highest education level, *% (n)*	
Left before GCSE	5.80 (17)
GCSE & Vocational	25.00 (73)
A-level	12.30 (36)
University	30.80 (90)
Postgraduate	26.00 (76)
Family income, *% (n)*	
<18,000	9.10 (27)
18–25,000	9.10 (27)
25–43,000	19.80 (59)
>43,000	50.00 (149)
Do not wish to say	12.10 (36)
Conception, *% (n)*	
Natural	95.70 (287)
Assisted	4.30 (13)
Smoking in pregnancy^a^, *% (n)*	
No	89.70 (270)
Yes	10.30 (31)
Alcohol in pregnancy^a^, *% (n)*	
No	65.20 (197)
Yes	34.80 (105)
Strenuous exercise, *% (n)*	
No	82.80 (250)
Yes	17.20 (52)
WIMD score^b^	1267.00 (1266.00)

BMI, Body Mass Index; GDM, Gestational diabetes mellitus; CBWC, Custom Birthweight Centile; SGA, Small-for-gestational age; AGA, Average-for-gestational age; LGA, Large-for-gestational age; WIMD, Welsh Index of Multiple Deprivation.

^a^At any point in pregnancy.

^b^WIMD score has a possible range of 1 to 1909, with a low score indicative of an area of higher deprivation and conversely a high score indicative of an area of lower deprivation.

#### Dietary patterns

Principal component analysis (PCA) with orthogonal varimax rotation was utilised to analyse the FFQ data. The scree plot, variance explained by each factor, factor loadings and subjective criteria (simplicity and interpretability) were utilised to determine the appropriate number of factors to derive. Solutions with differing numbers of factors were also assessed to determine suitability. Using this strategy, a solution with two factors was identified as best describing the dietary patterns in this study. The two dietary patterns explained 30.25% of the total variance within the data, the factor loadings of which are displayed in Table 2. Food items with factor loadings of ≥ 0.30 or ≤ -0.30 were considered to be strongly associated with the factor, and thus descriptive of that dietary pattern.

**Table 2:** Dietary patterns within the GiW cohort, identified by principal component analysis.

Food item	Western	Health conscious
Cakes/biscuits/icecream	**.71**	-.11
Chips/crisps	**.65**	-.21
Processed meat	**.54**	-.15
Takeout	**.54**	-.15
Chocolate	**.53**	-.05
Soft drinks	**.43**	-.21
Milk	**.41**	.27
Unprocessed meat	**.41**	.11
Bread/cereals/potatoes/rice/pasta	**.40**	.25
Caffeine	**.35**	.03
Salad/veg	-.04	**.76**
Dried fruit	-.11	**.69**
Fruit	-.10	**.60**
Supplements	-.13	**.49**
Meat alternatives	.02	**.45**
Fish/shellfish	-.04	**.43**
Cheese/yoghurt	.11	**.41**

The dietary patterns identified in Table 2 were labelled ‘Western’ and ‘Health conscious’ respectively. These labels were selected with consideration for existing literature investigating dietary patterns. The ‘Western’ dietary pattern explained 17.66% of variance and was characterised by high factor loadings on cakes/biscuits/icecream, chips/crisps, processed meat, takeout, chocolate, soft drinks, milk, unprocessed meat, bread/cereals/potatoes/rice/pasta and caffeine. The ‘Health conscious’ dietary pattern explained 12.59% of variance and was characterised by high factor loadings on salad/veg, dried fruit, fruit, supplements, meat alternatives, fish and cheese/yogurt.

#### Dietary patterns and associated variables

Analysis of variables potentially associated with each dietary pattern was undertaken utilising linear regression. At the univariate level (Table 3) maternal age, strenuous exercise and WIMD score were significantly negatively associated and categories within education and income as well as smoking in pregnancy were significantly positively associated with the ‘Western’ dietary pattern. Additionally maternal BMI, lower education categories, income and smoking in pregnancy were significantly negatively associated and maternal age, a postgraduate education and WIMD score were significantly positively associated with the ‘Health conscious’ dietary pattern.

**Table 3:** Univariate associations with GiW dietary patterns, identified by linear regression.

	‘Western’		‘Health conscious’	
	B	95% CI	B	95% CI
Fetal sex				
Female	*ref*		*ref*	
Male	.02	-.20, .25	.18	-.04, .41
Maternal BMI at booking	.01	-.01, .03	-.04***	-.06, -.02
Maternal age at booking	-.03**	-.05, -.01	.07***	.05, .09
Parity				
Multiparous	*ref*		*ref*	
Nulliparous	.16	-.13, .44	-.08	-.36, .20
Gestational weight gain (kg)	.00	-.02, .01	.00	-.01, .02
Gestational Diabetes (GDM)				
No	*ref*		*ref*	
Yes	-.11	-.63, .42	.27	-.25, .79
Highest education level				
Left before GCSE	.54**	.02, 1.06	-.66**	-1.14, -.19
GCSE & Vocational	.35**	.04, .66	-.76***	-1.04, -.47
A-level	.10	-.29, .49	-.73***	-1.08, -.37
University	*ref*		*ref*	
Postgraduate	-.06	-.37, .25	.30**	.02, .57
Family income				
<18,000	.16	-.25, .56	-.92***	-1.31, -.53
18–25,000	.42**	.02, .83	-.42**	-.81, -.03
25–43,000	-.02	-.32, .28	-.61***	-.90, -.32
>43,000	*ref*		*ref*	
Do not wish to say	.60**	.24, .96	.37**	-.72, -.02
Conception				
Natural	*ref*		*ref*	
Assisted	.04	-.52, .59	.09	-.46, .65
Smoking in pregnancy^a^				
No	*ref*		*ref*	
Yes	.43**	.06, .80	-.53**	-.90, -.16
Alcohol in pregnancy^a^				
No	*ref*		*ref*	
Yes	.02	-.22, .26	.01	-.22, .25
Strenuous exercise				
No	*ref*		*ref*	
Yes	-.31**	-.60, -.01	.59**	.29, .88
WIMD score	-2.68x10^-4^**	-4.44x10^-4^, -9.14x10^-5^	2.41x10^-4^**	6.54x10^-5^, 4.17x10^-4^

Ref, Reference; BMI, Body mass index; WIMD, Welsh Index of Multiple Deprivation.

^a^At any point in pregnancy.

** *p* < .05,

*** *p* < .001.

All variables significant at *p* < 0.05 at the univariate level were considered for inclusion in the adjusted multiple regression analysis. Multicollinearity was assessed via tolerance and VIF scores and found not to be present. At the multiple regression level (Table 4), no variables remained significantly associated with the ‘Western’ dietary pattern at a stringent cut-off of *p* = 0.05. However, maternal BMI, lower education categories and a category within income were significantly negatively associated and maternal age, a category within income and strenuous exercise were significantly positively associated with the ‘Health conscious’ dietary pattern.

**Table 4:** Associations with GiW dietary patterns, identified by multiple linear regression.

Variable	‘Western’		‘Health conscious’	
	AOR	95% CI	AOR	95% CI
Maternal BMI at booking	.01	-.02, .03	-.03**	-.05, -.01
Maternal age at booking	-.03*	-.06, .00	.05***	.03, .08
Highest education level				
Left before GCSE	.13	-.48, .74	-.56**	-1.08, -.03
GCSE & Vocational	.22	-.13, .57	-.64***	-.94, -.34
A-level	-.06	-.47, .35	-.55**	-.90, -.20
University	*ref*		*ref*	
Postgraduate	.04	-.27, .35	.15	-.12, .41
Family income				
<18,000	-.45*	-.97, .07	.03	-.42, .47
18–25,000	-.21	-.73, .31	.39*	-.06, .84
25–43,000	-.24	-.55, .08	-.32**	-.60, -.05
>43,000	*ref*		*ref*	
Do not wish to say	.34	-.10, .78	.20	-.18, .58
Smoking in pregnancy^a^				
No	*ref*		*ref*	
Yes	.33	-.11, .77	-.26	-.64, .13
Strenuous exercise				
No	*ref*		*ref*	
Yes	-.20	-.51, .12	.38***	.10, .65
WIMD score	-1.72x10^-4^	-3.82x10^-4^, 3.80x10^-5^	-1.50x10^-4^	-3.31x10^-4^, 3.30x10^-4^

AOR, Adjusted odds ratio; Ref, Reference; BMI, Body mass index; WIMD, Welsh Index of Multiple Deprivation.

AOR = the unstandardised coefficient B

^a^At any point in pregnancy.

* *p* < .10,

** *p* < .05,

*** *p* < .001.

### Dietary patterns and custom birthweight centiles (CBWC)

Multiple linear regression was utilised to investigate the association between dietary patterns and CBWC (Table 5). The unadjusted analysis identified that only the ‘Health conscious’ dietary pattern was significantly associated with CBWC (*p* = < 0.001). This significant association remained when the analysis was adjusted for potential confounding variables. Specifically, for each 1 unit increase in ‘Health conscious’ dietary pattern score, CBWC increased by 4.75.

**Table 5:** Unadjusted and adjusted multiple linear regression: The association between dietary patterns and CBWC.

Model	Dietary pattern	*P*	B	95% CI
Unadjusted	Western	.109	-2.64	-5.87, .59
	Health Conscious	< .001	5.81	2.58, 9.04
Adjusted*	Western	.297	-1.76	-5.07, 1.55
	Health Conscious	.010	4.75	1.17, 8.33

*Maternal age, smoking during pregnancy, alcohol during pregnancy, exercise, GDM, conception & WIMD score.

### Dietary patterns and classification of size for gestational age

To further understand the relationship between dietary patterns and infant birthweight, the association between dietary patterns and SGA and LGA infants was investigated. Separate analyses were undertaken for SGA and LGA, with both compared to AGA. Unadjusted binary logistic regression between dietary patterns and SGA compared to AGA revealed only the ‘Health conscious’ dietary pattern to be significantly associated (Table 6). This significant association remained when adjusted for potential confounding variables, with a 1 S.D increase in ‘Health conscious’ dietary pattern score reducing the odds of delivering an SGA infant compared to an AGA infant by a factor of 0.51. Binary logistic regression was also utilised to investigate the association between dietary patterns and LGA compared to AGA (Table 6). Neither dietary pattern was found to be significantly associated with LGA, unadjusted or adjusted.

**Table 6:** Unadjusted and adjusted binary logistic regression: Association between dietary patterns and SGA compared to AGA and LGA compared to AGA.

	Model	Dietary pattern	*P*	β	95% CI
SGA	Unadjusted	Western	.142	1.38	.90, 2.13
		Health Conscious	.002	.45	.27, .75
	Adjusted*	Western	.850	1.06	.61, 1.84
		Health Conscious	.046	.51	.26, .99
LGA	Unadjusted	Western	.780	.96	.69, 1.32
		Health Conscious	.099	1.33	.95, 1.86
	Adjusted*	Western	.849	1.04	.72, 1.50
		Health Conscious	.228	1.29	.85, 1.94

* Maternal age, smoking during pregnancy, alcohol during pregnancy, exercise, GDM, conception & WIMD score.

## Discussion

The aim of this study was to investigate dietary patterns during pregnancy and their association with infant birthweight outcomes utilising CBWC in a cohort of women living in Wales. Two dietary patterns were identified within this cohort: ‘Western’ and ‘Health conscious’. Higher adherence to the ‘Health conscious’ dietary pattern was associated with higher maternal age, undertaking strenuous exercise and an income of £18–25,000, whereas lower adherence was associated with higher maternal BMI, lower educational attainment and an income of £25–43,000. A greater adherence to the ‘Health conscious’ dietary pattern was associated with increased CBWC, or higher birthweight, and a significantly reduced risk of delivering an SGA infant.

Due to the population-specific nature of dietary patterns that are driven by data, making comparisons to other published research is challenging. Nevertheless, despite differences in the naming of dietary patterns, similarities can be identified within the dietary pattern characteristics. The ‘Health conscious’ dietary pattern in the current study is consistent with versions of healthy or prudent dietary patterns identified in numerous studies investigating dietary patterns [38, 39], including that found in a large UK based cohort study [27]. Additionally, the ‘Western’ dietary pattern in the current study relates to unhealthy dietary patterns in previous literature, including the ‘Snack’ and ‘Processed’ patterns within another UK-based study [26] and the ‘Western’ dietary pattern identified within a large Australian cohort [40]. However, differences can be seen when considering [29] and [30], based in Finland and Norway respectively, especially when regarding more ‘traditional’ patterns influenced by culture, highlighting the population-specific nature of dietary patterns.

The identified association between greater adherence to the ‘Health conscious’ dietary pattern, increased birthweight and reduced risk of delivering an SGA infant in the GiW cohort is also supported by existing literature. A comprehensive review by [32] concluded that dietary patterns characterised by high intakes of vegetables, fruits and dairy products were associated with reduced risk of SGA. Similarly [31] identified that high adherence to a ‘Mediterranean’ dietary pattern reduced the risk of a growth restricted infant in terms of weight, although the specific dietary pattern differed between regions in Spain and Greece. Moreover, whilst not an investigation of specific dietary patterns, [41] concluded that greater adherence to the Alternative Healthy Eating Index was related to reduced SGA risk. Interestingly, [34] identified that in white Europeans a plant-based dietary pattern was associated with lower birthweight and increased SGA risk, highlighting the influence ethnicity may have in this area. However, our results differ from the conclusions drawn in the [32] review in that we did not observe that the unhealthier dietary pattern was associated with decreased birthweight or increased SGA risk. This may be due to our use of CBWC, the methodology utilised to measure diet or specific characteristics of our population.

CBWC are a relatively recent introduction to studies on birthweight despite being recommended in the UK by the Royal College of Obstetricians and Gynaecologists since 2002 [42]. We were able to find only one published study exploring maternal diet in relation to CBWC that had been adjusted for various maternal and infant characteristics [31], which reported a higher adherence to a meditaranean diet reduced the risk of delivering a growth restricted infant and increased birthweight only in mothers who smoked. It will be important to review existing data in the context of this more accurate approach to classifying infants.

Diet was ascertained using a FFQ which provide a longer-term representation of diet during the pregnancy compared with other commonly utilised self-report methods, such as 24 hour dietary recalls [24]. Participants are required to reflect on their diet throughout the pregnancy rather than for a shorter specific period. While the FFQ was completed at only one timepoint, [28] demonstrated that dietary patterns do not change significantly throughout pregnancy, and thus one time point accurately reflects the pregnancy period. However, a potential limitation of our methodology is the relatively small size of our FFQ with 17 items. The questionnaire was evaluated by an experienced dietician to ensure that it could simply assess diet through a sufficient range of food items, reviewed for suitability and successfully piloted. Nevertheless, the FFQ has not currently been formally validated and a more extensive questionnaire might have increased sensitivity. Even so, we were still able to distinguish distinct patterns and associations with birth outcomes.

A second limitation is the size of our target population. Following the exclusion criteria, data of 303 participants from the 355 recruited was analysed. While small, the study was highly focused including only women recruited the morning prior to an ELCS by two research midwives with singleton pregnancies and no known infection or fetal anomaly. Moreover, we analysed data from only Caucasian participants, constituting 91% of the cohort recruited, to minimise the heterogeneity which can introduce potential confounders or ‘noise’ in smaller studies, negatively influencing results [43]. While this focus overcomes some of the limitations of a small study size, it does mean that our findings may not be applicable to other modes of delivery or ethnicities in Wales. These limitations should be taken into account when considering the reported association between the ‘Health conscious’ dietary pattern and reduced SGA risk. Nonetheless, this is still an important population to study as white Welsh are considered a distinct ethnicity in the UK [44] and the global incidence of caesarean sections (CS) has nearly doubled in recent years [45, 46]. In Wales alone in 2015–2016 there were 30,254 deliveries of which 26% were by caesarean with 11.8% being elective procedures [47]. Despite this high and increasing incidence of CS we could not identify any other research investigating dietary patterns and birthweight in this population. Moreover, whilst this was a relatively well-educated population with a high level of family income, there was still a range of education and income levels present, which potentially reduces the issue of representativeness of the overall Welsh population. Further explorations in a larger sample will be required to elucidate the presence or absence of the reported associations in the wider population.

Whilst studies have investigated dietary patterns in pregnancy, both alone and in relation to birthweight, no existing studies were identified that reported on maternal dietary pattern in Wales, despite the differences in healthcare to other UK nations. Moreover, existing studies rarely utilise CBWC despite the advantages of this method. The current study identified two dietary patterns within the GiW cohort; ‘Western’ and ‘Health conscious’. A healthy diet was found to be associated with higher CWBC, or increased birthweight, and reduced risk of delivering an SGA infant. This might suggest a protective role against poor infant outcomes. More should be done to highlight the link between healthy diet and heathy infant birthweight in Wales, with focused behavioural interventions in areas of Wales encouraging healthier dietary habits to improve pregnancy outcomes for both infant and mother.

## Supporting information

S1 Table Comparison of the demographic data of those included (n = 303) and excluded in the study (n = 45) with the exception of participants who withdrew. (DOCX)
