# Supplementary material for: The Grown in Wales Study: Examining dietary patterns, custom birthweight centiles and the risk of delivering a small-for-gestational age (SGA) infant
Source: PLoS One. 2019 Mar 12;14(3):e0213412. doi: 10.1371/journal.pone.0213412 (PMC6413917; doi:10.1371/journal.pone.0213412)
Supplement: S1 Table — (DOCX) [file pone.0213412.s001.docx]

**Supplement 1 – Comparison of the demographic data of those included (n=303) and excluded in the study (n=45) with the exception of participants who withdrew, utilising Mann Whitney U for continuous data and Chi Square for categorical data.**

|  | Included (n = 303) | Excluded (n = 45) |  |
| --- | --- | --- | --- |
| Demographics | % (n) or median (IQR) | % (n) or median (IQR) | *p* |
| Maternal BMI at booking | 26.17 (7.42) | 26.99 (6.81) | .701 |
| Maternal age at booking | 33.00 (6.00) | 31.00 (8.50) | .160 |
| Parity, *% (n)* |  |  |  |
| Multiparous | 79.50 (241) | 90.90 (40) | .073 |
| Nulliparous | 20.50 (62) | 9.10 (4) |  |
| Gestational weight gain (kg) | 14.85 (7.88) | 14.70 (10.50) | .407 |
| GDM |  |  |  |
| Yes | 95.00 (285) | 88.40 (38) | .083 |
| No | 5.00 (15) | 11.60 (5) |  |
| Fetal sex, *% (n)* |  |  |  |
| Female | 54.10 (164) | 58.50 (24) | .594 |
| Male | 45.90 (139) | 41.50 (17) |  |
| CBWC | 58.60 (48.90) | 56.90 (54.00) | .880 |
| Size for gestational age *% (n)* |  |  |  |
| SGA | 7.30 (22) | 4.90 (2) | .573 |
| AGA | 78.20 (237) | 85.40 (35) |  |
| LGA | 14.50 (44) | 9.80 (4) |  |
| Highest education level, *% (n)* |  |  |  |
| Left before GCSE | 5.80 (17) | 5.30 (2) | .172 |
| GCSE & Vocational | 25.00 (73) | 13.20 (5) |  |
| A-level | 12.30 (36) | 7.90 (3) |  |
| University | 30.80 (90) | 50.00 (19) |  |
| Postgraduate | 26.00 (76) | 23.70 (9) |  |
| Family income, *% (n)* |  |  |  |
| <18,000 | 9.10 (27) | 10.30 (4) | .036 |
| 18 – 25,000 | 9.10 (27) | 17.90 (7) |  |
| 25-43,000 | 19.80 (59) | 12.80 (5) |  |
| >43,000 | 50 (149) | 33.30 (13) |  |
| Do not wish to say | 12.10 (36) | 25.60 (10) |  |
| Conception, *% (n)* |  |  |  |
| Natural | 95.70 (287) | 95.00 (38) | .847 |
| Assisted | 4.30 (13) | 5.00 (2) |  |
| Smoking in pregnancy^a^, *% (n)* |  |  |  |
| No | 89.70 (270) | 90.20 (37) | .914 |
| Yes | 10.30 (31) | 9.80 (4) |  |
| Alcohol in pregnancy^a^, *% (n)* |  |  |  |
| No | 65.20 (197) | 85.40 (35) | .010 |
| Yes | 34.80 (105) | 14.60 (6) |  |
| Strenuous exercise, *% (n)* |  |  |  |
| No | 82.80 (250) | 92.50 (37) | .116 |
| Yes | 17.20 (52) | 7.50 (3) |  |
| WIMD score^b^ | 1267.00 (1266.00) | 746.50 (1017.50) | .009 |

BMI, Body Mass Index; GDM, Gestational diabetes mellitus; CBWC, Custom Birthweight Centile; SGA, Small-for-gestational age; AGA, Average-for-gestational age; LGA, Large-for-gestational age; WIMD, Welsh Index of Multiple Deprivation
^a^At any point in pregnancy
